# Fault Recovery Strategy with Net Load Forecasting Using Bayesian Optimized LSTM for Distribution Networks

**DOI:** 10.3390/e27090888

**Published:** 2025-08-22

**Authors:** Zekai Ding, Yundi Chu

**Affiliations:** College of Artificial Intelligence and Automation, Hohai University, Nanjing 210024, China; zekaiding@hhu.edu.cn

**Keywords:** active distribution network, fault recovery, LSTM, Bayesian optimization, GA-QPSO

## Abstract

To address the impact of distributed energy resource volatility on distribution network fault restoration, this paper proposes a strategy that incorporates net load forecasting. A Bayesian-optimized long short-term memory neural network is used to accurately predict the net load within fault-affected areas, achieving an R^2^ of 0.9569 and an RMSE of 12.15 kW. Based on the forecasting results, a fast restoration optimization model is established, with objectives to maximize critical load recovery, minimize switching operations, and reduce network losses. The model is solved using a genetic algorithm enhanced with quantum particle swarm optimization (GA-QPSO), a hybrid metaheuristic known for its superior global exploration and local refinement capabilities. GA-QPSO has been successfully applied in various power system optimization problems, including service restoration, network reconfiguration, and distributed generation planning, owing to its effectiveness in navigating large, complex solution spaces. Simulation results on the IEEE 33-bus system show that the proposed method reduces network losses by 33.2%, extends the power supply duration from 60 to 120 min, and improves load recovery from 72.7% to 75.8%, demonstrating enhanced accuracy and efficiency of the restoration process.

## 1. Introduction

With the rapid development of renewable energy, the large-scale integration of DG presents new challenges to the stable operation of distribution networks, especially under the carbon strategy of carbon peaking and carbon neutrality. To enhance the grid’s self-healing capability following faults while ensuring supply–demand balance and maintaining power quality, it is essential to design efficient fault restoration strategies based on accurate net load forecasting [1]. In this context, fault restoration strategies that combine islanding operation with network reconfiguration have emerged as a prominent research focus, owing to their ability to flexibly reallocate power and rapidly restore critical loads [2].

Extensive research has been conducted worldwide on fault restoration in distribution networks with a high penetration of DG. Reference [3] proposes a fault restoration strategy tailored for DG-integrated systems and introduces two islanding operation criteria. Building on this, Ref. [4] quantifies the influence of weather conditions on component failure rates and suggests leveraging DG to enhance grid resilience. However, this method fails to precisely characterize the supply–demand balance between DG output and load demand, potentially resulting in DG instability and energy curtailment [5].

To address computational efficiency, Ref. [6] introduces an automatic fast search strategy, significantly improving solution performance. Nonetheless, it requires predefined fault scenarios and lacks dynamic adaptability. In response, Ref. [7] adopts cloud-edge collaborative technology in flexible interconnected distribution networks to enhance the real-time responsiveness of fault restoration algorithms. Ref. [8] proposes a new fast self-healing technology for distribution networks based on line topology and beat frequency attenuation, using strategies like network reconfiguration, IIDG control and redundant resource modeling, with advantages of fast response, high recovery rate and reduced loss. Similarly, Ref. [9] incorporates photovoltaic (PV) forecasting results into a dynamic tree knapsack model to construct an optimal fault restoration strategy. Commonly used fault restoration algorithms include classical methods [10,11] and nature-inspired heuristic optimization approaches. Classical algorithms often suffer from local optima and are increasingly inadequate for modern power systems. Nature-inspired heuristic optimization approaches, such as ant colony optimization [12,13], firefly algorithms [14,15], and particle swarm optimization (PSO) [16,17], have gained attention for their superior global search capabilities. Among these algorithms, genetic algorithms have been widely applied in fault restoration strategies due to their population-based exploration and flexible encoding schemes. However, GAs may still converge prematurely to suboptimal solutions. To address this issue, Ref. [18] proposes the integration of QPSO to perform secondary refinement of GA-generated solutions, thereby improving convergence accuracy and solution quality in complex restoration scenarios.

In the domain of load forecasting, AI-based models frequently employ techniques such as backpropagation neural networks (BPNNs) [19,20], convolutional neural networks (CNNs) [21,22], and recurrent neural networks (RNNs) [23]. Among them, long short-term memory (LSTM) networks have been extensively adopted for load and renewable energy forecasting, owing to their superior ability to capture temporal dependencies in time series data. Nevertheless, the performance of LSTM models is highly sensitive to hyperparameter configurations such as learning rate, batch size, and number of hidden units. This makes hyperparameter optimization a critical aspect of LSTM model design, warranting in-depth investigation to improve prediction accuracy.

In summary, current research on fault restoration in distribution networks has made notable progress. However, under the growing integration of distributed energy resources, existing approaches often fall short in accurately capturing net load characteristics and ensuring timely restoration. This may result in power imbalances and voltage violations during network reconfiguration.

To address the aforementioned challenges, this study proposes an efficient fault restoration strategy that integrates islanding and network reconfiguration, based on GA-QPSO, with full consideration of net load forecasting results. First, a short-term net load forecasting method is developed using an LSTM neural network, whose hyperparameters—such as learning rate, batch size, and number of hidden units—are optimized using Bayesian techniques to enhance predictive accuracy (LSTM–Bayesian). Based on the forecasting output, a mathematical model for fault restoration is formulated. Then, a hybrid GA-QPSO algorithm is employed to optimize the islanding configuration. Specifically, the GA is first used to perform global exploration and generate candidate solutions, which are subsequently refined by QPSO to perform local exploitation and identify high-quality optimal solutions, thereby improving convergence stability and precision. Finally, simulations are conducted on the IEEE 33-bus distribution system with integrated distributed generation to validate the accuracy and effectiveness of the proposed strategy.

## 2. Net Load Forecasting Based on Bayesian-Optimized LSTM

### 2.1. Long Short-Term Memory Network

**Figure 1:**
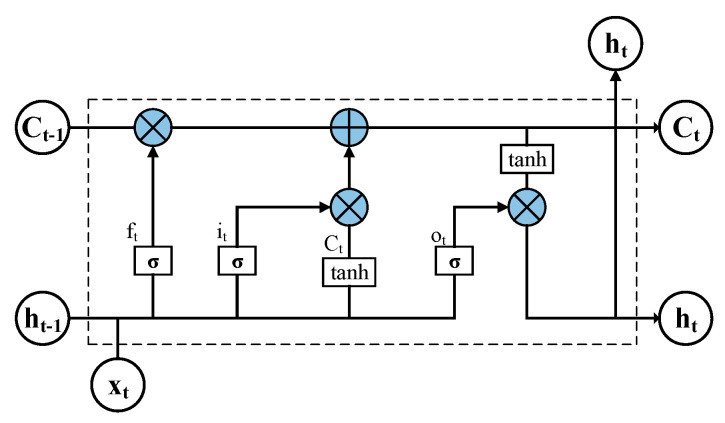
Structure of the long short-term memory network.

The long short-term memory network is an improved variant of the recurrent neural network (RNN) [24]. By introducing a gating mechanism, LSTM enables the selective retention and forgetting of information within sequences, demonstrating significant advantages in time series forecasting, natural language processing, speech recognition, and related fields. In power system applications, LSTM is widely used for load forecasting as well as photovoltaic (PV) and wind power prediction due to its ability to capture temporal dependencies and periodic patterns. The structure of the LSTM network is illustrated in Figure 1.

The LSTM network consists of three types of gates: the input gate, output gate, and forget gate. The input gate determines when new information should be written into the memory cell, the output gate controls the information to be sent out from the cell, and the forget gate decides which historical information should be discarded. The mathematical expressions of these three gates are given in Equations (1)–(3), respectively:

(1) $$f_{t}=\sigma (W_{f}\cdot [h_{t-1},x_{t}]+b_{f})$$

(2) $$i_{t}=\sigma (W_{i}\cdot [h_{t-1},x_{t}]+b_{i})$$

(3) $$o_{t}=\sigma (W_{o}[h_{t-1},x_{t}]+b_{o})$$

where the components of the equations are as follows:

- $W_{f}$, $W_{i}$, and $W_{o}$ are the weight matrices associated with the forget gate, input gate, and output gate, respectively;
- $b_{f}$, $b_{i}$, and $b_{o}$ are the corresponding bias vectors;
- $x_{t}$ denotes the input vector at time step t;
- $h_{t-1}$ is the output (hidden state) from the previous time step;
- σ represents the sigmoid activation function;
- $f_{t}$, $i_{t}$, and $o_{t}$ refer to the forget gate, input gate, and output gate activations at time t, respectively.

The candidate memory cell uses the hyperbolic tangent (tanh) function as its activation, and the retained information is computed as shown in Equation (4):

(4) $$\bar{C_{t}}=\tanh(W_{c}\cdot [h_{t-1},x_{t}]+b_{C})$$

where $W_{c}$ denotes the weight matrix, $b_{C}$ is the bias vector, and $\bar{C_{t}}$ represents the candidate memory cell state reflecting the retained information.

The update of the cell state is calculated as shown in Equation (5):

(5) $$C_{t}=f_{t}*C_{t-1}+i_{t}*\bar{C_{t}}$$

where ∗ denotes the element-wise (Hadamard) multiplication, and $C_{t}$ represents the updated cell state.

The hidden state is calculated as in Equation (6):

(6) $$h_{t}=o_{t}*\tanh(C_{t})$$

When the output gate activation approaches 1, the LSTM network effectively transmits all retained information to the prediction module. Through gating mechanisms, LSTM selectively preserves important information over long time horizons while discarding irrelevant data, which is particularly crucial for tasks requiring long-term dependencies.

### 2.2. Bayesian Optimization Algorithm

The core strength of Bayesian optimization lies in its ability to address two major challenges in hyperparameter tuning for machine learning models: the uncertainty of the objective function and the high computational cost of evaluating it. By constructing a probabilistic surrogate model, Bayesian optimization updates the posterior distribution of the objective function based on a limited number of evaluations. This iterative process efficiently guides the search toward the optimal set of hyperparameters with significantly reduced computational overhead [25].

As an efficient global optimization strategy, the core architecture of the Bayesian optimization algorithm consists of two key components: the probabilistic surrogate model and the acquisition function, which work in tandem to guide the search. The execution process is as follows:

1. Initialization: Construct a prior distribution for the surrogate model, serving as the initial approximation of the objective function.
2. Sampling point selection: Determine the next sampling point x by maximizing the acquisition function a(x), which balances the trade-off between exploring uncertain regions and exploiting areas with known promising results.
3. Objective evaluation: Evaluate the objective function c(x) at the selected point x, and obtain the corresponding output value y.
4. Model update: Update the surrogate model using the newly observed data pair (x,y), resulting in a revised posterior distribution that reflects the most recent information.
5. Iterative optimization: Repeat steps 2–4 until a stopping criterion is met, such as reaching the maximum number of iterations or satisfying convergence conditions.

In practice, Gaussian Processes (GPs) are the most widely used probabilistic surrogate models in Bayesian optimization, owing to their flexible non-parametric nature and strong capability for uncertainty quantification. The acquisition function, on the other hand, guides the search process by determining where to sample next. Common acquisition functions include Probability of Improvement (PI) and Expected Improvement (EI). These functions are designed to balance two competing goals: (1) local exploitation, by refining the search near the current best-known solution to identify potentially better candidates; and (2) global exploration, by sampling in less-explored regions to avoid premature convergence and enhance the global search capability of the algorithm.

### 2.3. Bayesian Optimization of LSTM

The performance of LSTM is highly sensitive to the configuration of their hyperparameters. However, the nonlinear interactions and interdependencies among these parameters make the tuning process challenging. Prior studies have demonstrated that the optimal selection of key hyperparameters—such as the number of hidden neurons, learning rate, and batch size—can substantially enhance the predictive accuracy of LSTM models in time series forecasting tasks [26].

To enhance the accuracy of net load forecasting, this paper proposes the use of Bayesian optimization for tuning the hyperparameters of LSTM networks. In power system load forecasting and renewable generation prediction scenarios, Bayesian optimization efficiently identifies optimal hyperparameter combinations by constructing probabilistic surrogate models—such as Gaussian Processes—and updating the posterior distribution of the objective function using historical evaluation data. Through acquisition functions like Expected Improvement (EI), it achieves a balance between exploration and exploitation with minimal evaluations. This approach significantly improves LSTM’s ability to model load fluctuations and generation uncertainties, effectively reduces forecasting errors, provides a more reliable basis for fault recovery strategies, and contributes to the efficient and stable operation of power systems.

To address different levels of missing data in the samples, this paper adopts a tiered preprocessing strategy. For short gaps (fewer than 10 consecutive missing time steps), linear interpolation is employed for data imputation. For moderate gaps (between 10 and 100 missing steps), a moving average method is used. In cases of extensive missing data (exceeding 100 time steps), the affected samples are discarded entirely. Furthermore, if more than 20% of the values in a dataset are missing, the entire dataset is excluded from analysis.

To quantitatively evaluate the performance of the logging curve prediction, the Root Mean Square Error (RMSE) and the coefficient of determination (R^2^) are adopted as evaluation metrics. Their calculation formulas are given in Equations (7) and (8):

(7) $$RMSE=\sqrt{\frac{1}{N}\sum_{i=1}^{N}{\left({\hat{y}}_{i}-y_{i}\right)}^{2}}$$

(8) $$R^{2}=\frac{\left(N\sum xy-\sum x\sum y\right)}{\sqrt{\left[(N\sum x^{2}-{\left(\sum x\right)}^{2})\times (N\sum y^{2}-{\left(\sum y\right)}^{2})\right]}}$$

where N is the total number of data points, ${\hat{y}}_{i}$ denotes the predicted value for the i data point, and $y_{i}$ represents the corresponding true value.

**Figure 2:**
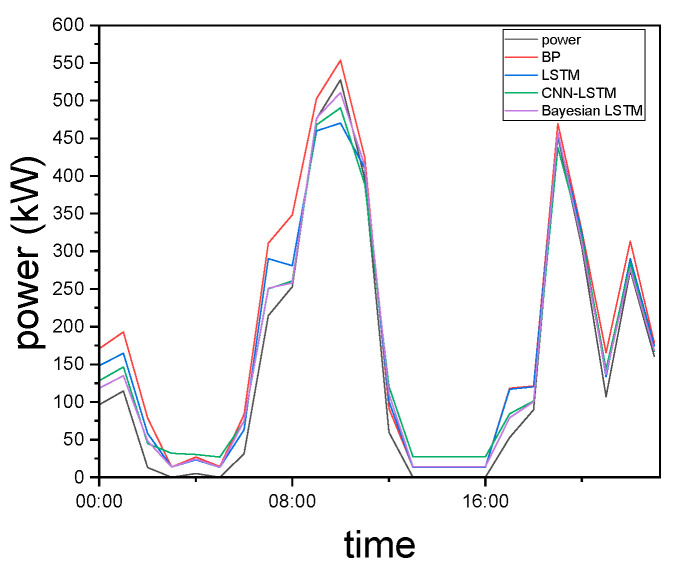
Comparison of prediction performance across different algorithms.

In this paper, measured wind power data from [27] are used to compare the prediction results for a typical day. The comparison is illustrated in Figure 2 and summarized in Table 1.

The results clearly demonstrate that the Bayesian optimization–LSTM model achieves superior peak prediction accuracy compared to other models and outperforms the CNN-LSTM model in valley prediction. In terms of overall performance, the Bayesian optimization–LSTM exhibits a significant improvement in prediction accuracy, as measured by RMSE, and achieves higher feature correlation, as reflected by the R^2^ value, compared to other commonly used models.

The optimization model is ultimately applied to forecasting by integrating meteorological and power generation datasets. Specifically, meteorological data from days with similar weather conditions are selected, with the power generation data from the day prior to each meteorologically similar day used as the input and the generation data on the similar day itself used as the output. This approach enables the training of a DG output forecasting model, which can be similarly applied to load prediction.

After obtaining the forecasts for power output and load, the net load is calculated using Equation (9):

(9) $$P_{net}=\sum P_{load}-\sum P_{DG}$$

where $P_{net}$ denotes the net load power, $\sum P_{load}$ denotes the total load demand, and $\sum P_{DG}$ denotes the total output power of distributed generation.

## 3. Fault Recovery Strategies for Distribution Networks with Distributed Generation

### 3.1. Objective Function

After a distribution network fault occurs, the load power supply in the non-faulted areas is first restored through main grid reconfiguration. Subsequently, an objective function is formulated to restore power to the remaining unserved areas via islanding. The objective function is defined as shown in Equation (10):

(10) $$f_{\min}=\omega_{1}\cdot \sum_{i=1}^{n}\sum_{j=1}^{3}Z_{j}P_{i}+\omega_{2}\cdot \sum_{a=1}^{L}\left|K_{a}-K_{a}^{*}\right|+\omega_{3}\cdot \sum_{k=1}^{s}\beta_{k}R_{k}\frac{P_{k}^{2}+Q_{k}^{2}}{U_{k}^{2}}$$

where the components of the equation are as follows:

- $\sum_{i=1}^{n}\sum_{j=1}^{3}Z_{j}P_{i}$ denotes the total power of different classes of de-energized loads;
- $\sum_{a=1}^{L}\left|K_{a}-K_{a}^{*}\right|$ denotes the number of switching operations;
- $\beta_{k}R_{k}(P_{k}^{2}+Q_{k}^{2})/U_{k}^{2}$ denotes the active power loss in the network.

Additional notation is as follows:

- n is the total number of load nodes without power;
- $Z_{j}$ denotes the class of load at node j;
- $P_{i}$ is the active power of the $i^{th}$ important lost load node;
- L is the total number of switches in the system;
- $K_{a}$ and $K_{a}^{*}$ represent the switching states of switch iii before and after reconfiguration, respectively, where 1 indicates closed and 0 indicates open;
- s is the total number of branches in the system;
- $\beta_{k}$ is the switching state of branch k, where 1 indicates closed and 0 indicates open;
- $R_{k}$ is the resistance of branch k;
- $P_{k}$ and $Q_{k}$ are the active and reactive power flows on branch k;
- $U_{k}$ is the voltage magnitude at the receiving end of branch k,

In addition, $\omega_{1}$, $\omega_{2}$, $\omega_{3}$ are weighting coefficients reflecting the priority of the objectives: prioritizing restoration of important loads and minimizing power loss are core objectives, while the number of switching operations and line losses are conventional objectives. Therefore, the weighting values must be assigned according to their priority levels.

Specifically, the weights are assigned as follows:

For the first set of objectives: $Z_{1}$:$Z_{2}$:$Z_{3}$ = 100:10:1;

For the second set of objectives: $\omega_{1}$:$\omega_{2}$:$\omega_{3}$ = 7:1:2.

### 3.2. Model Constraints

(1) Nodal Power Balance Constraint:

(11) $$\left\{\begin{array}{l} P_{Gi}+P_{DGi}-P_{Li}=U_{i}\sum_{j=1}^{N}U_{j}(G_{ij}\cos\theta_{ij}+B_{ij}\sin\theta_{ij})\\ Q_{Gi}+Q_{DGi}-Q_{Li}=U_{i}\sum_{j=1}^{N}U_{j}(G_{ij}\cos\theta_{ij}+B_{ij}\sin\theta_{ij}) \end{array}\right.$$

where $P_{Gi}$ and $Q_{Gi}$ denote the active and reactive power injected by node i respectively. $P_{DGi}$ and $Q_{DGi}$ denote the active and reactive power injected by the DG of node i, respectively. $P_{Li}$ and $Q_{Li}$ denote the active and reactive loads of node i, respectively. $U_{i}$ and $U_{j}$ denote the voltages at the first and last nodes i and j of the branch, respectively. $G_{ij}$ and $B_{ij}$ denote the conductance and conductivity between the $i^{th}$ and $j^{th}$ nodes, respectively, and $\theta_{ij}$ denotes the phase difference between the $i^{th}$ and $j^{th}$ nodes.

(2) Nodal voltage constraints:

(12) $$U_{i}^{\min}\leq U_{i}\leq U_{i}^{\max}$$

where $U_{i}^{\min}$ and $U_{i}^{\max}$ denote the minimum and maximum allowable voltages at node i.

(3) Branch active power constraints:

(13) $$|P_{i}|\leq P_{i}^{\max}$$

where $P_{i}$ is the active power of branch i and $P_{i}^{\max}$ is the maximum allowable active power for branch i.

(4) Distributed generation constraints:

(14) $$P_{DGi}^{\min}\leq P_{DGi}\leq P_{DGi}^{\max}$$

where $P_{DGi}^{\min}$ and $P_{DGi}^{\max}$ denote the minimum and maximum generation power of the distributed generator at node i.

### 3.3. Islanding and Network Reconfiguration Recovery Based on GA-QPSO Algorithm

GA is an intelligent optimization technique inspired by the principles of biological evolution, renowned for its strong global search capability and parallel processing features [28]. In distribution networks, where a large number of switches are involved—especially under large-scale faults or outages—the resulting solution space for reconfiguration and islanding becomes significantly large and complex. GA demonstrates clear advantages in such scenarios, as it effectively handles the intricate topological constraints of power systems and efficiently explores the vast search space. However, conventional GAs often suffer from limited local search accuracy, which can lead to premature convergence to suboptimal solutions during the evolutionary process, ultimately affecting the quality and robustness of the final optimization results.

To overcome the aforementioned limitations, this paper introduces and enhances the quantum-behaved particle swarm optimization (QPSO) algorithm. QPSO incorporates probabilistic behavioral models from quantum mechanics, offering superior local search accuracy and a stronger ability to escape local optima. This makes it an effective complement to the GA, particularly in compensating for GA’s insufficient local exploitation capability. By integrating QPSO into the GA framework and applying fine tuning after each generation of evolution, the overall optimization accuracy and convergence speed are significantly improved. This hybrid approach markedly enhances the global search performance, solution stability, and robustness of the recovery strategy. The mathematical formulations of the QPSO algorithm are presented in Equations (15)–(17):

(15) $$p_{i}=\frac{pbest_{i}+gbest}{2}$$

(16) $$mbest=\frac{1}{N}\sum_{i=1}^{N}pbest_{i}$$

(17) $$x_{i}(t+1)=p_{i}\pm \beta \cdot |mbest-x_{i}(t)|\cdot \ln(\frac{1}{u})$$

where $pbest_{i}$ represents the historical optimal position of the particle itself; gbest represents the global optimal position; $p_{i}$ is the center of the search direction; mbest represents the average of the current historical optimal positions of all particles; β represents the contraction–expansion factor; $x_{i}(t+1)$ represents the position of the next moment; and u is a random number obeying the [0, 1] uniform distribution.

The objectives of islanding and restorative reconfiguration include minimizing network losses, reducing the number of switching operations, minimizing load shedding, and prioritizing critical load restoration. The procedure for islanding and restorative reconfiguration is outlined as follows:

1. Initial data input: Import the post-fault distribution network data, including topology, fault location, load distribution, and switching states, where each switch node is binary-encoded (0/1) to represent on/off status.
2. Feasible solution generation and population initialization: Construct an initial solution set meeting network constraints using a radial structure strategy, ensuring reasonable network distribution and a stable base for optimization.
3. Genetic evolution and structural optimization: Apply selection, crossover, and mutation operations to globally explore the solution space, iteratively updating islanding schemes to maintain population diversity and solution feasibility.
4. Local search enhancement: Introduce QPSO for the local optimization of elite individuals after each genetic generation, leveraging quantum behavior and the best population positions to improve convergence and escape local optima.
5. Islanding scheme verification: Assess the optimized islanding solutions for physical topology, load distribution, and voltage stability and analyze potential power imbalances using load forecasts.
6. Dynamic topology update: Update the network topology based on optimization results to clarify backbone connectivity and available branches for subsequent reconfiguration.
7. Population regeneration and iterative optimization: Use loop coding and genetic recombination to regenerate feasible solutions, evaluate them via the fitness function, and retain the best individuals to guide evolution.
8. Final recovery scheme determination: Perform gradient-level fine tuning on the optimal solution to maximize load restoration, network connectivity, and operational stability.

**Figure 3:**
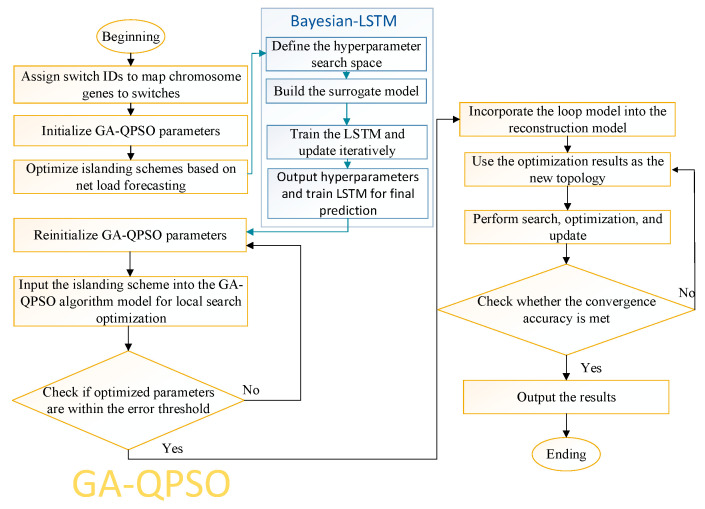
Algorithm flowchart of the proposed GA-QPSO fault recovery method.

The algorithm flowchart of the proposed method is illustrated in Figure 3.

## 4. Validating Cases

### 4.1. Results and Analyses

**Figure 4:**
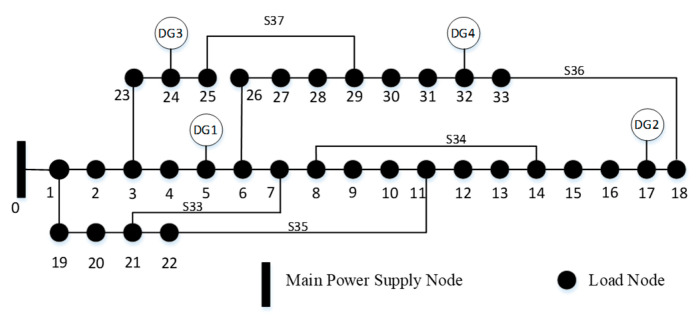
Architecture diagram of the IEEE 33-bus distribution system.

To assess the effectiveness of the proposed reconfiguration and islanding recovery strategy, the IEEE 33-bus distribution system—depicted in Figure 4—is adopted as the test case. This widely used benchmark system consists of 33 buses, 32 sectionalizing switches, and 5 tie switches and operates at a nominal voltage level of 12.66 kV.

The load categories and their corresponding weight factors for each bus are presented in Table 2, while the locations and capacities of distributed generation units are detailed in Table 3.

**Figure 5:**
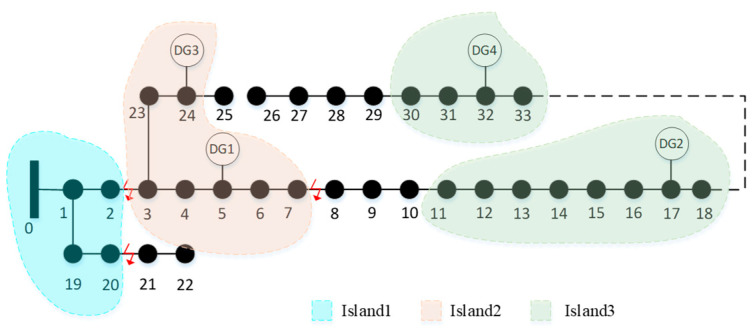
Preliminary siloing scheme diagram.

Assuming natural disasters cause multiple line outages on lines 2–3, 7–8, and 20–21 between 10:00 and 12:00 on a given day, the system is partitioned into three silos according to the silo division scheme, as illustrated in Figure 5. The GA-QPSO algorithm parameters are set as follows: population size of 100, chromosome length of 36, stochastic variance of 0.15, maximal GA iterations of 50, maximal QPSO iterations of 30, particle number of 20, and a convergence control parameter of 1.5. These values were determined based on empirical tuning through multiple experimental trials. The parameters were adjusted iteratively to balance optimization quality and computational efficiency, ensuring stable convergence behavior for the IEEE 33-bus test system.

**Figure 6:**
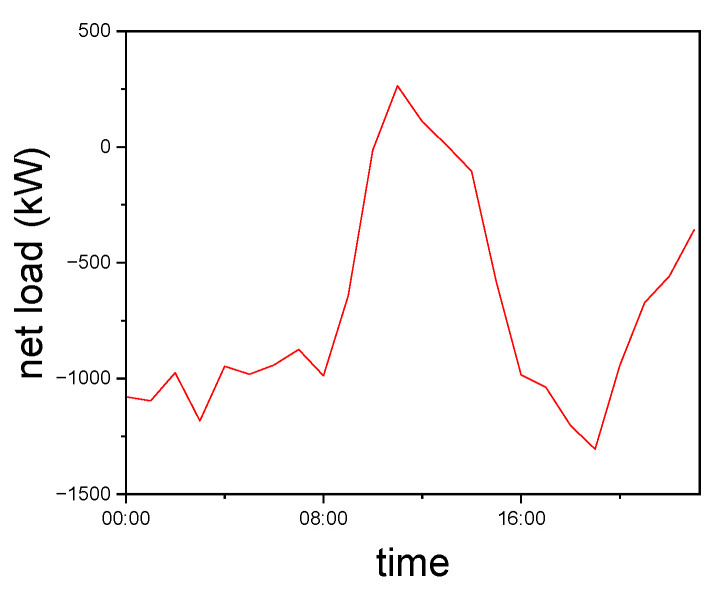
Net load forecast results.

Power supply to Island 1 can be restored through main network reconfiguration. For the remaining unenergized islands, planned islands are formed by utilizing DGs. Based on the preliminary division results, there is an option to close certain tie switches to merge DG2 and DG4 into a larger Island 3. Therefore, accurate load forecasting is necessary to support this decision. In this study, net load forecasting for the isolated islands is performed using data from [27], with the results presented in Figure 6.

**Figure 7:**
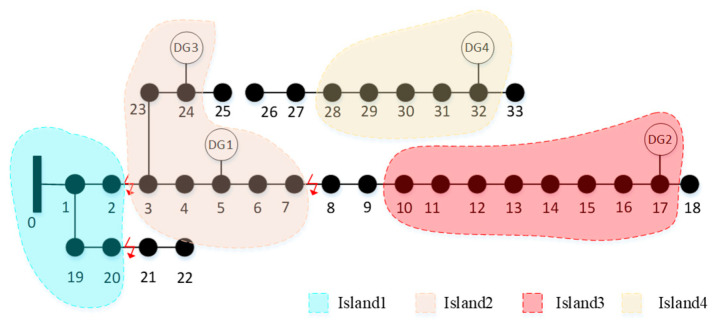
Final restoration strategy diagram.

Based on the net load prediction results, it is evident that merging DG2 and DG4 into a larger island for fault recovery poses a safety risk, since the net load remains positive between 11:00 and 13:00 on that day. Therefore, this option is excluded. The updated network topology is then subjected to local optimization using the GA-QPSO algorithm with the same parameter settings as previously described. The final islanding and restoration configuration is illustrated in Figure 7.

As shown in Figure 7, the reconfiguration restores power supply to nodes 10, 28, and 29.

**Figure 8:**
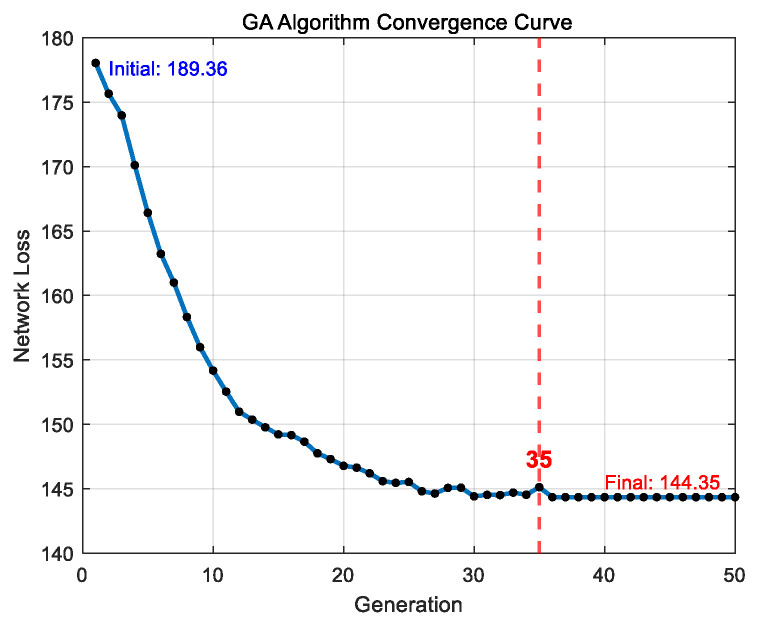
GA convergence curve diagram.

**Figure 9:**
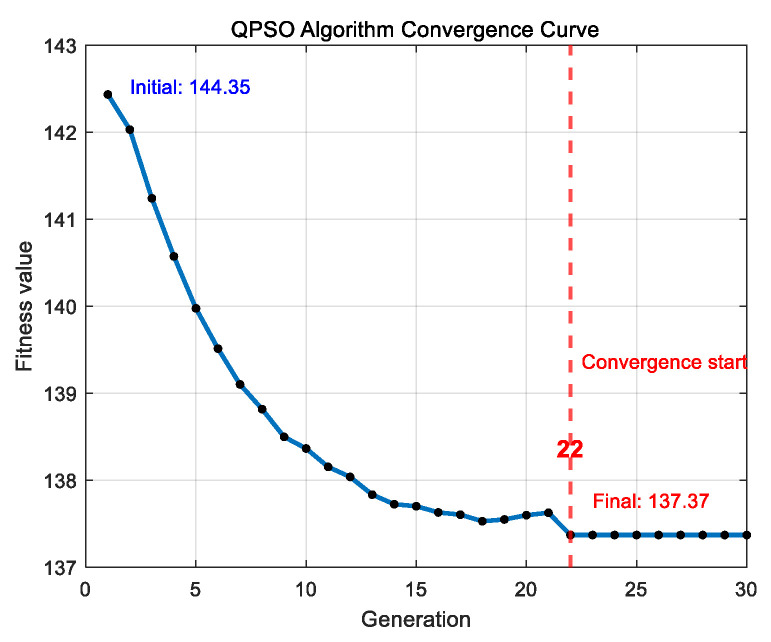
QPSO convergence curve diagram.

As shown in Figure 8 and Figure 9, the reconfiguration successfully restores power supply to nodes 10, 28, and 29. The optimization results are summarized in Table 4. Specifically, the GA begins to converge after 35 iterations, with the fitness value decreasing from 189.36 to 144.35. Subsequently, the quantum-behaved particle swarm optimization, initialized with the GA solution, starts converging after 22 iterations, further reducing the fitness value to 137.37.

The optimization results are summarized in Table 4.

The results indicate that the proposed algorithm effectively reduces network losses. The switching operations are limited to two actions within the permissible range, and the method requires only 7 s to execute. Specifically, this computation time refers to the duration from the completion of the island partition optimization to the generation of the final fault recovery strategy, including the execution of the GA-QPSO optimization process. With its fast response characteristics, the approach can significantly enhance power grid stability.

### 4.2. Performance Comparison

**Figure 10:**
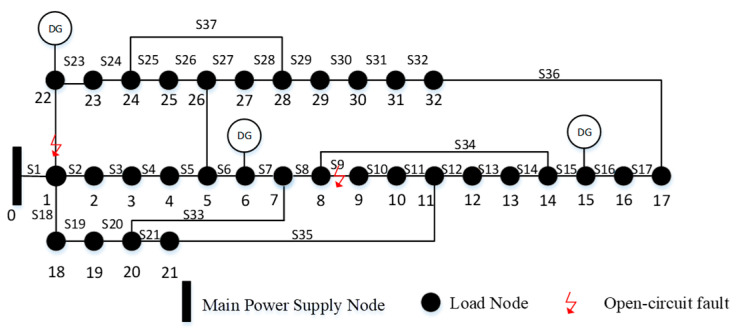
Structure of the IEEE 33-node distribution network as a benchmark case.

To validate the superiority of the operational algorithms proposed in this paper for distribution network fault recovery, the IEEE 33-node system with three DGs, adapted from references [29,30], is selected as the benchmark case, as shown in Figure 10. In this system, node 0 represents the power source. It is assumed that permanent faults occur at sectional switches S9 and S22. The pre-fault recovery state, the recovery strategies from [29,30], and the strategy proposed in this paper are compared with the results summarized in Table 5.

It can be observed that both the method proposed in [28] and the recovery strategy presented in this paper demonstrate improved performance compared to the pre-fault state. However, the strategy proposed herein achieves recovery with fewer switching operations (2 vs. 3) while maintaining lower network losses (76.08 kW vs. 118.51 kW in [29] and 71.08 kW in [30]), making it more conducive to optimizing the objective function.

This improvement can be attributed to the hybrid design of the GA-QPSO algorithm. The GA ensures global exploration of the vast reconfiguration space, while the QPSO refines high-quality individuals through local exploitation, thus avoiding premature convergence and local optima. Additionally, the integration of real-time net load forecasting allows for more adaptive and secure islanding decisions, especially in scenarios involving fluctuating distributed generation. This ensures that the final configuration avoids unsafe operations such as merging DGs when the net load remains positive, as discussed in Section 4.1.

In contrast, the strategies in [29] and [30] either rely on rule-based heuristics or fixed topological assumptions, which may limit flexibility or responsiveness to actual net load conditions. The proposed method, by contrast, dynamically incorporates forecast information into the reconfiguration process, enabling more accurate recovery with minimal actions. These advantages highlight its suitability for real-time self-healing in modern distribution networks.

## 5. Conclusions

In this paper, a comprehensive strategy is proposed to address the dynamic fault recovery problem in active distribution networks, integrating net load forecasting, islanding operation management, and network reconfiguration optimization. A corresponding mathematical recovery model is developed. The experimental results demonstrate that the GA-QPSO algorithm reduces network losses by 33.2% within only 7 s of computation time. Furthermore, the Bayesian-optimized LSTM model improves prediction accuracy on the test set by 46.3% compared to the conventional LSTM benchmark. This approach significantly enhances the real-time response capability of distribution network fault recovery and improves system operational stability.

Despite its promising performance, the proposed strategy has several limitations. The forecasting module depends on high-quality and complete meteorological and generation datasets, which may be unavailable in some practical environments. Additionally, while the GA-QPSO algorithm is computationally efficient in medium-sized networks, its scalability in large-scale systems with higher DG penetration remains to be further validated. Moreover, ideal communication infrastructure and complete system observability are assumed, which may not always hold true in actual applications.

Future work will focus on incorporating stochastic forecasting and robust optimization to improve adaptability under uncertainty. Communication delays, partial observability, and real-time dispatch constraints will also be considered to enhance the practical feasibility of the approach. Furthermore, the method will be tested on larger-scale distribution systems and integrated into realistic operation platforms. Engineering scenarios such as those presented in [31], which utilize real-world distribution network data from Beijing and focus on post-disaster emergency resource coordination, provide valuable references and practical environments for the future validation of our proposed strategy. We will further seek cooperation with the authors of that study and request the dataset to verify our method. With the advancement of the “Carbon Peak and Carbon Neutrality” goals, further research will also explore incorporating green optimization metrics such as carbon emission cost and energy efficiency into the multi-objective framework.

## Figures and Tables

**Table 1:** Comparison of prediction performance across different algorithms.

Algorithm	R^2^	RMSE/kW
BP	0.7963	36.3064
LSTM	0.8951	22.7879
CNN-LSTM	0.9193	17.7116
Bayesian–LSTM	0.9569	12.1464

**Table 2:** Nodal load category.

Load Category	Weighting Factor	Respective Buses
Category I load	100	3, 10, 11, 24, 31
Category II load	10	4, 6, 12, 15, 19, 21, 22, 23, 29, 30
Category III load	1	2, 5, 7, 8, 9, 13, 14, 16, 17, 18, 20, 25, 26, 27, 28, 32, 33

**Table 3:** Distributed generation connection buses and their capacities.

Connection Node	Typology	Active Power Capacity/kW
5	photovoltaic	750
17	wind power	600
24	photovoltaic	500
32	wind power	720

**Table 4:** Pre-optimization vs. post-optimization performance.

Comparison Metrics	Pre-Optimization	Post-Optimization
Operating switch	-	Disconnect line segments 25–29 and 18–33
Number of operations	-	2
Network loss /kW	122.0886	81.5665
Minimum voltage/pu	0.9759	0.9759
Load recovery percentage	72.7%	75.8%
Power supply duration /min	60	120
Increased electricity supply/kWh	-	81.0442

**Table 5:** Comparison of restoration strategies.

Restoration Strategy	Disconnect Switch	Number of Switching Operations	Network Loss/kW
**Before fault recovery**	S9, S22, S35–S37	0	95.35
**Literature** [29]	S9, S12, S14, S23, S33, S35–S37	3	118.51
**Literature** [30]	S9, S22, S17, S23, S33, S35–S37	3	71.08
**Methodology of this paper**	S9, S10, S16, S20, S23, S35–S37	2	76.08

## Data Availability

The original contributions presented in this study are included in the article. Further inquiries can be directed to the corresponding author.

## Abbreviations

The following abbreviations are used in this manuscript:

DG	Distributed Generation
QPSO	Quantum-behaved Particle Swarm Optimization
GA	Genetic Algorithm
LSTM	Long Short-Term Memory network
GA-QPSO	Genetic Algorithm and Quantum-behaved Particle Swarm Optimization
RNN	Recurrent Neural Network
